# Protein quality of insects as potential ingredients for dog and cat foods*

**DOI:** 10.1017/jns.2014.23

**Published:** 2014-09-25

**Authors:** Guido Bosch, Sheng Zhang, Dennis G. A. B. Oonincx, Wouter H. Hendriks

**Affiliations:** 1 Animal Nutrition Group, Wageningen University, PO Box 338, 6700 AH Wageningen, The Netherlands; 2 Laboratory of Entomology, Wageningen University, PO Box 8031, 6700 EH Wageningen, The Netherlands

**Keywords:** Dog, Cats, Nutritional value, Amino acid composition, *In vitro* digestibility, AA, amino acid, CP, crude protein, OM, organic matter

## Abstract

Insects have been proposed as a high-quality, efficient and sustainable dietary protein source. The present study evaluated the protein quality of a selection of insect species. Insect substrates were housefly pupae, adult house cricket, yellow mealworm larvae, lesser mealworm larvae, Morio worm larvae, black soldier fly larvae and pupae, six spot roach, death's head cockroach and Argentinean cockroach. Reference substrates were poultry meat meal, fish meal and soyabean meal. Substrates were analysed for DM, N, crude fat, ash and amino acid (AA) contents and for *in vitro* digestibility of organic matter (OM) and N. The nutrient composition, AA scores as well as *in vitro* OM and N digestibility varied considerably between insect substrates. For the AA score, the first limiting AA for most substrates was the combined requirement for Met and Cys. The pupae of the housefly and black soldier fly were high in protein and had high AA scores but were less digestible than other insect substrates. The protein content and AA score of house crickets were high and similar to that of fish meal; however, *in vitro* N digestibility was higher. The cockroaches were relatively high in protein but the indispensable AA contents, AA scores and the *in vitro* digestibility values were relatively low. In addition to the indices of protein quality, other aspects such as efficiency of conversion of organic side streams, feasibility of mass-production, product safety and pet owner perception are important for future dog and cat food application of insects as alternative protein source.

Trends towards 2050 predict an increased demand for animal-derived protein sources for human foods due to the combined effects of human population increase and increasing standards of living in developing countries^(^^1^^)^. This demand will increase the global competition for proteins in human food, pet food and livestock feed and stimulate the development of alternative and sustainable protein sources for assuring food security. The Food and Agricultural Organization of the United Nations has highlighted the potential of insects as food and feed sources^(^^2^^)^. Insects are in general proteinaceous^(^^3^^)^ and some species can be efficiently grown on organic side streams making these potentially sustainable alternatives for current proteinaceous feed ingredients^(^^3^^–^^5^^)^. In addition, insects are commonly consumed by feral cats around the world contributing up to 6 % of their diet^(^^6^^)^. The information on the protein quality is, however, currently limited for most insect species. The aim of the present study was, therefore, to evaluate the protein quality of a selection of insect species as potential ingredients for dog and cat foods.

## Experimental methods

### Substrates

Insect substrates were housefly pupae (*Musca domestica*) (donated by Jagran B. V. Hillegom), adult house cricket (*Acheta domesticus*), yellow mealworm larvae (*Tenebrio molitor*), lesser mealworm larvae (*Alphitobius diaperinus*), Morio worm larvae (*Zophobas morio*) (all purchased from Kreca), black soldier fly (*Hermetia illucens*) larvae and pupae (donated by Laboratory of Entomology, Wageningen University) and adult six spot roach (*Eublaberus distanti*), adult death's head cockroach (*Blaberus craniifer*) and adult female Argentinean cockroach (*Blaptica dubia*) (donated by D. G. A. B. Oonincx). The black soldier fly larvae were fed a broiler starter diet (Agruniek Rijnvallei Voer BV) and the roaches were fed household food waste. The other insect species were sourced from companies that keep the diet compositions confidential. Reference substrates were poultry meat meal (Sonac), fish meal (Research Diet Services) and soyabean meal (Research Diet Services). Housefly pupae, black soldier fly larvae, and pupae and cockroaches were freeze-dried to a constant weight. House crickets, yellow mealworms, lesser mealworms and Morio worms were already freeze-dried. Remaining poultry manure attached to the housefly pupae and dirt attached to black soldier fly pupae were removed by hand. Before milling, housefly pupae, Morio worms, black soldier fly larvae and pupae, and cockroaches were broken using an ultracentrifugal mill without a sieve (Retsch ZM 100, F. Kurt Retsch GmbH& Co. KG). Then these insects were ground using a laboratory analytical mill (A10, Janke & Kunkel GmbH u. Co KG), except for house crickets that were ground in centrifugal mill with a 1 mm sieve (Retsch ZM 100). Reference substrates were already in a dried and ground form.

### *In vitro* digestion

Substrates were *in vitro* digested according to an up-scaled Boisen two-step method^(^^7^^)^ with modifications^(^^8^^,^^9^^)^ simulating the canine gastric and small intestinal digestive processes. Chloramphenicol was added during incubation for its antibiotic effect. The number of replicate incubations required was calculated on the anticipated amount of residue per replicate and the total amount of residue required for chemical analyses. Substrates (10 g) were incubated in beakers with a phosphate buffer solution (250 ml, 0·1 m, pH 6·0) and an HCl solution (100 ml, 0·2 m). The pH was adjusted to 2·0 with 1 m HCl or 10 m NaOH. Fresh pepsin solution (10 ml, 25 g/l, porcine pepsin 2000 FIP U/g, Merck 7190) and 10 ml chloramphenicol solution (0·005 g/mol ethanol) were added and each beaker was covered with a glaze and placed in a heating chamber at 39°C for 2 h under constant magnetic stirring. Then, 90 ml phosphate buffer (0·2 m, pH 6·8) and 50 ml of a 0·6 m NaOH were added into the solution. The pH was adjusted to 6·8 with 1 m HCl or 10 m NaOH. Fresh pancreatin solution (10 ml, 100 g/l pancreatin, Porcine pancreas grade VI, SigmaP-1750) was added and incubation was continued for 4 h under the same conditions. After incubation, the residues were collected by filtration of the slurries on a nylon gauze (37 µm) folded in a Büchner porcelain funnel. The sample was washed twice with acetone (99·5 %) followed by ethanol (96 %). Then the cloth with the residue was temporarily placed on a clean paper to evaporate the remaining ethanol/acetone overnight. The residue was collected from the nylon cloth and dried at 70°C overnight in a preweighed jar. Then the oven-dried jars were reweighed to determine the amount of dry residue for each replicate, which allowed the calculation of DM digestibility for each replicate. For each type of substrate, the selected oven-dried residues were pooled and ground in laboratory analytical mill (A10, Ika-Werk). The ground residues were transferred into a new jar, pending further chemical analyses for calculating the *in vitro* DM, organic matter (OM) and N digestibility for each substrate.

### Chemical analyses

DM and ash were determined by drying to a constant weight at 103°C and combusting at 550°C, respectively. Nitrogen was determined using the Kjeldahl method^(^^10^^)^, and crude fat was analysed according to the Berntrop method^(^^11^^)^. Amino acids (AA) were analysed by ion exchange chromatography and ninhydrin derivatisation^(^^12^^)^.

### Calculations

OM content was calculated at the 100 – ash content (percentage of DM). Crude protein (CP) was calculated as 6·25 × N and AA content was expressed as percentage of CP. Digestibility of substrate OM and N was calculated as the amount of residue collected (in g DM) × content in residue (in percentage of DM basis)/amount of substrate incubated (in g DM) × content in substrate (in percentage of DM basis). The AA scores were calculated as described in Kerr *et al.*^(^^13^^)^ using minimal requirements for growth of kittens and puppies^(^^14^^)^ as reference values.

## Results and discussion

Protein and fat contents varied considerably between insect substrates (Table 1). The CP content of insect substrates was in general higher than that in soyabean meal and close to that in poultry meat meal and fish meal. House crickets contained the most CP followed by lesser mealworms and the roaches. Fat content ranged from 12·8 to 39·6 % of DM for black soldier fly larvae and Morio worms, respectively. Crude ash content of insect substrates was between 3·0 and 5·6 % of DM, except for the black soldier fly larvae and pupae containing about 13 %. Ash contents of black soldier fly larvae ranged in literature from 9·0 to 14·6 % of DM^(^^15^^,^^16^^)^ and 15·5 % of DM in prepupae^(^^17^^)^. Phe and Met contents of CP varied the most between insect substrates, with highest contents found for the housefly pupae. Housefly pupae were also high in Lys as were the lesser mealworms. House crickets were relatively high in Arg but low in His. As it has been suggested that CP approximates the true protein for most species of insects^(^^18^^)^, the AA were expressed on a CP basis to gain insight in the protein quality. Chitin contributes to non-protein N and contributes 1–7 % of the whole-body N^(^^18^^)^. Differences in chitin content of insect substrates may confound the estimation of protein quality. AA contents for insect species vary considerably among studies. For example, for house crickets, Arg content in the present study (5·7 % of CP) was within the range of other studies^(^^3^^)^ (4·9–6·0 % of CP) but His was higher (3·4 *v.* 2·1–2·6 % of CP). Depending on the diet fed, Met content of yellow mealworms ranged from 0·48 to 1·80 % of CP^(^^19^^)^. For application of insects as a protein source in pet food or feed, it would be of importance to monitor and control the variation in AA composition. Met and Cys in poultry meat meal was lower in the present study than reported in the literature, i.e. 1·05 *v.* 1·07 % in Clapper *et al.*^(^^20^^)^ to 2·11 % in Johnson *et al.*^(^^21^^)^ and 0·69 % (data not shown) *v.* 1·34 % in Clapper *et al.*^(^^20^^)^ to 2·66 % in Murray *et al.*^(^^22^^)^, respectively. For the AA score, the first limiting AA for most substrates was the combined requirement for Met and Cys. Highest AA scores were found for housefly pupae, followed by black soldier fly pupae and Morio worm and lowest scores for the cockroaches.

**Table 1.** Proximate composition (percentage of DM), indispensable amino acid composition (percentage of CP) and amino acid (AA) score of insect and reference substrates

	Insect substrates										Reference substrates		
Parameter	HFp	BSFl	BSFp	HC	YMW	LMW	MW	SSR	DHCR	ACR*	PMM	FM	SBM
CP	62·5	56·1	52·1	70·6	52·0	64·8	47·0	66·3	65·0	64·4	69·1	71·0	51·6
Fat	19·2	12·8	19·7	17·7	33·9	22·2	39·6	25·1	22·0	24·5	12·8	9·2	2·5
Ash	5·6	12·6	13·9	5·3	3·9	4·1	3·0	3·6	3·9	4·4	15·4	19·9	6·8
*AA*													
Arg	4·2	3·7	4·2	5·7	4·6	4·8	4·6	3·6	3·9	3·5	5·8	4·5	6·3
His	4·8	4·4	4·7	3·4	5·1	4·9	4·8	4·3	4·6	4·5	3·7	3·4	3·1
Ile	4·0	4·0	4·2	4·0	4·6	4·6	5·0	3·4	3·7	3·2	3·8	4·8	5·0
Leu	6·1	6·1	6·5	6·6	7·3	6·7	7·2	5·4	5·9	5·3	6·4	7·1	7·8
Lys	6·2	5·4	5·4	5·8	5·5	6·5	5·3	4·3	4·7	4·0	5·6	7·4	6·2
Met	2·6	1·4	1·7	1·6	1·4	1·3	1·6	1·3	1·2	1·3	1·0	1·9	2·0
Phe	5·2	3·1	3·3	3·2	3·4	3·9	3·7	2·6	2·7	2·7	3·3	3·5	5·2
Thr	3·8	3·6	3·6	3·6	4·0	4·0	4·1	3·1	3·3	3·1	3·6	4·0	3·9
Val	5·0	5·5	5·7	5·7	6·3	5·9	6·5	5·6	6·1	5·4	4·6	5·0	5·0
tIAA	41·8	37·1	39·3	39·6	42·3	42·7	42·7	33·5	36·2	33·1	37·8	41·5	44·4
*AA scores*†													
Dog	94·0	63·4	74·4	69·3	68·4	60·4	73·8	53·0	55·5	59·7	44·6	73·1	89·1
Cat	106·1	79·2	93·0	86·6	85·5	75·5	92·2	66·2	69·4	74·6	55·8	91·6	107·5

CP, crude protein; HFp, housefly pupae; BSFl and BSFp, black soldier fly larvae and pupae; HC, house cricket; YMW, yellow mealworm; LMW, lesser mealworm; MW, Morio worm; SSR, six spot roach; DHC, death's head cockroach; ACR, Argentinean cockroach; PMM, poultry meat meal; FM, fish meal; SBM, soyabean meal; tIAA, total indispensable amino acids.

*Females.

†Calculated as described in Kerr *et al.*^(^^13^^)^ using minimal requirements for growth of kittens and puppies^(^^14^^)^ as reference values.

*In vitro* OM digestibility was highest for yellow mealworms, Morio worms and lesser mealworms (Table 2). Black soldier fly pupae had lowest *in vitro* OM digestibility and was 16·2 % lower than for the larvae. This difference in digestibility is likely caused by a higher cuticular protein-sclerotisation in the pupae. *In vitro* N digestibility was relatively high for the house crickets, yellow mealworms, lesser mealworms and Morio worms and low for black soldier fly pupae, six spot roach and death's head cockroach. Information on the digestibility of evaluated insect species is limited in the literature. Apparent faecal N digestibility of a diet containing 33 % black soldier fly larvae meal as the main protein source was 76·0 % in 8·2–14·7 kg barrows^(^^16^^)^ and a diet containing 50 % housefly pupae meal had an apparent faecal N digestibility of 79·0 % in broilers^(^^23^^)^.

**Table 2.** *In vitro* digestibility (%) of insect and reference substrates

	Digestibility	
Substrate	OM	N
*Insect*		
Housefly pupae	83·2	84·3
BSF larvae	84·3	89·7
BSF pupae	68·1	77·7
House cricket	88·0	91·7
Yellow mealworm	91·5	91·3
Lesser mealworm	90·2	91·5
Morio worm	91·1	92·0
Six spot roach	77·8	76·4
Death's head CR	79·4	78·4
Argentinean CR*	84·0	83·8
*Reference*		
Poultry meat meal	85·8	87·9
Fish meal	82·1	85·7
Soyabean meal	80·6	94·7

OM, organic matter; BSF, black soldier fly; CR, cockroach.

*Females.

Selected insect substrates differed considerably in nutrient composition as well as *in vitro* OM and N digestibility. Of the insect substrates studied, the pupae of the housefly and black soldier fly were high in CP and had high AA scores but were less digestible than the other insect substrates. The CP content and AA score of house crickets were high and similar to that of fish meal but with slightly higher *in vitro* N digestibility. The cockroaches were relatively high in CP but the indispensable AA contents, the AA scores and *in vitro* digestibility values were relatively low. Next to these indices of protein quality, other aspects such as efficiency of conversion of organic side streams^(^^2^^,^^24^^)^, feasibility of mass-production^(^^24^^)^, product safety^(^^24^^,^^25^^)^ and pet owner perception will determine if insect species are used in future pet food formulations. These and other aspects require further study.
